# Reliability of Protective Coatings for Flexible Piezoelectric Transducers in Aqueous Environments

**DOI:** 10.3390/mi10110739

**Published:** 2019-10-31

**Authors:** Massimo Mariello, Francesco Guido, Vincenzo Mariano Mastronardi, Roberto Giannuzzi, Luciana Algieri, Antonio Qualteri, Alfonso Maffezzoli, Massimo De Vittorio

**Affiliations:** 1 Istituto Italiano di Tecnologia, Center for Biomolecular Nanotechnologies, 73010 Arnesano (Lecce), Italy; francesco.guido@iit.it (F.G.); vincenzo.mastronardi@iit.it (V.M.M.); roberto.giannuzzi@iit.it (R.G.); antonio.qualtieri@iit.it (A.Q.); massimo.devittorio@iit.it (M.D.V.); 2 Dipartimento di Ingegneria dell’Innovazione, Università del Salento, 73100 Lecce, Italy; alfonso.maffezzoli@unisalento.it; 3 Piezoskin s.r.l., 73010 Arnesano (Lecce), Italy; lalgieri@piezoskin.com

**Keywords:** waterproof, coating, reliability, flexible micro-devices, piezoelectric transducers, aqueous environments, seawater

## Abstract

Electronic devices used for marine applications suffer from several issues that can compromise their performance. In particular, water absorption and permeation can lead to the corrosion of metal parts or short-circuits. The added mass due to the absorbed water affects the inertia and durability of the devices, especially for flexible and very thin micro-systems. Furthermore, the employment of such delicate devices underwater is unavoidably subjected to the adhesion of microorganisms and formation of biofilms that limit their reliability. Thus, the demand of waterproofing solutions has increased in recent years, focusing on more conformal, flexible and insulating coatings. This work introduces an evaluation of different polymeric coatings (parylene-C, poly-dimethyl siloxane (PDMS), poly-methyl methacrylate (PMMA), and poly-(vinylidene fluoride) (PVDF)) aimed at increasing the reliability of piezoelectric flexible microdevices used for sensing water motions or for scavenging wave energy. Absorption and corrosion tests showed that Parylene-C, while susceptible to micro-cracking during prolonged oscillating cycles, exhibits the best anti-corrosive behavior. Parylene-C was then treated with oxygen plasma and UV/ozone for modifying the surface morphology in order to evaluate the biofilm formation with different surface conditions. A preliminary characterization through a laser Doppler vibrometer allowed us to detect a reduction in the biofilm mass surface density after 35 days of exposure to seawater.

## 1. Introduction

One of the most crucial engineering issues regarding the application of micro and nanoscale electronic devices in liquid environments is the need of being protected from the external harsh surroundings. This is mainly due to the risk of short-circuit caused by the absorption and permeation of water, which is generally also responsible of degradation of devices made of layered functional structures [1,2,3]. In this respect, delamination is one of the major modes of failure of organic and inorganic layered systems and consists of the weakening or loss of adherence between the different layers, resulting from mechanical strain mismatches or electrochemical reactions at the interfaces [4]. Moreover, the contact between water (especially salty water when dealing with marine applications) and the metal parts inside the micro-systems leads in most cases to spread or localized corrosion of electrodes and wires, degrading the electronic conduction and the device intrinsic performance, as a consequence [5,6,7]. In addition, an unavoidable phenomenon related to the submersion of devices underwater, is the accumulation of micro-organisms, such as bacteria or unicellular algae, on their surface, namely the biofouling, which is characterized by a gradual and persistent formation of slimy biofilms [8,9,10,11,12,13,14]. 

**Figure 1:**
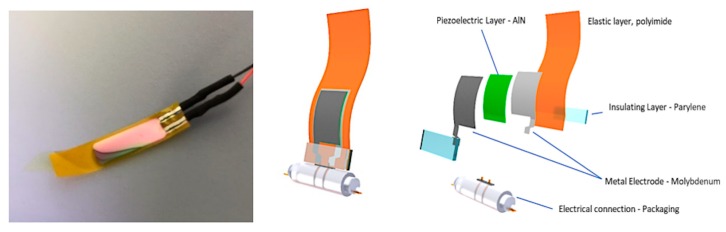
Photo and expanded view of the layered structure of the first group of piezoelectric micro-devices employed for energy harvesting in fluid environments. The thin films were deposited by reactive sputtering, as described in [15,16].

For applications in aqueous environments, electronic micro-systems must therefore be protected with watertight electrically insulating coatings. Nowadays, the miniaturization of devices, the discovery of new lightweight materials as substrates and the adoption of advanced microfabrication techniques, have led to the design and fabrication of novel flexible devices of cm-size with components of the order of 0.1 mm. They are generally based on stacking sequences of several functional thin-film layers. In this work we focused in particular on the reliability of micro-transducers for harvesting mechanical energy from fluid flows. Two sample categories were selected. The first one embodies transducers made of kapton as substrate, covered by a piezoelectric stack deposited by reactive sputtering and patterned by lithography and etching techniques. The expanded view of the layered structure is reported in Figure 1: here, the active region consists of an aluminum nitride (AlN) piezoelectric layer sandwiched between two thin molybdenum (Mo) electrodes. 

**Figure 2:**
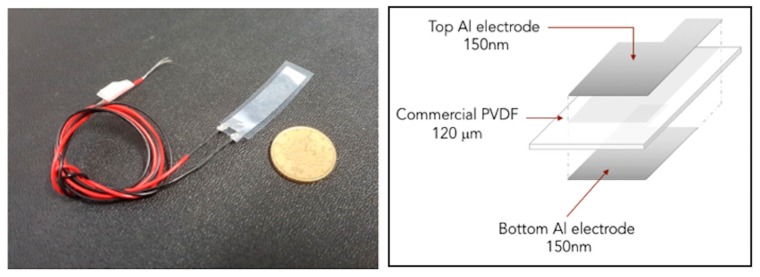
Photo and schematic of the second group of micro-devices. The thin electrodes were deposited by thermal evaporation using shadow masks.

The second sample group includes unimorph devices with a simpler structure made of a poly-(vinylidene fluoride), (PVDF), bi-axially oriented piezoelectric foil sandwiched between two thin-film aluminum (Al) electrodes, as illustrated in Figure 2.

**Figure 3:**
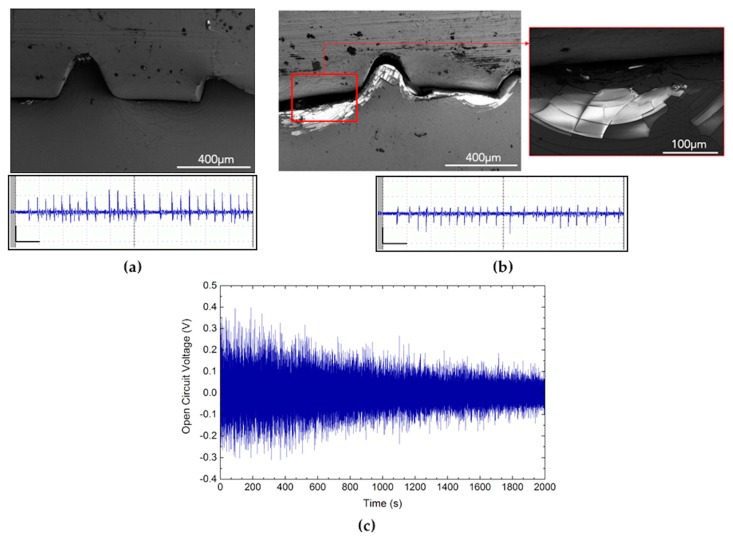
Scanning electron microscope (SEM) images showing crack growth before (**a**) and after (**b**) long-term utilization in correspondence of local defect points, i.e., the areas where the electrical crimp terminals are inserted. The output signals (peak-to-peak voltage) show a decrease in performance due to crack propagation; scale bars: 1.0 s (horizontal), 200 mV (vertical). In short-lasting periods, i.e., after submersion, the decreasing signal amplitude is due to sudden defects, such as delaminations (**c**).

These devices are aimed at undergoing low-frequency fluid-induced oscillations in a single-cantilever-beam configuration. The purpose is to scavenge energy which would be otherwise lost (harvesters) or to sense water motions (sensors), thus they should continuously work for long-lasting periods without decrease in performances [17]. As an example, Figure 3a,b report scanning electron microscope (SEM) images of some defects generated during a long working period of an AlN-based device at the points of crimped regions, whereas Figure 3c shows the general decreasing in output signal after the sudden exposure to seawater: it is worth noticing that after removing and drying the devices, their performances come back unaltered although the presence of defects, such as delaminations grown in short-testing period, was found.

**Figure 4:**
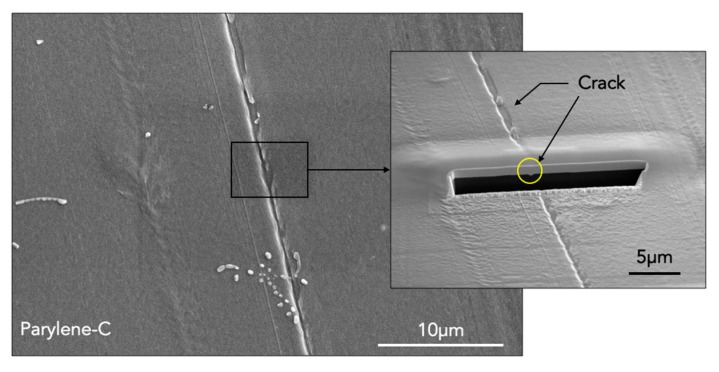
SEM micrograph: flexible kapton substrate coated with thin-film parylene after oscillatory movements in long-term period. The inset shows a cross section made by focused ion beam (FIB) in correspondence of the surface crack. The yellow circle indicates that the crack does not go deep inside up to the substrate.

Due to continuous oscillatory movement of the flexible devices, some cracks, growing from surface defects, propagate inside the film and they reach eventually the active region of the device, therefore affecting its performance. Figure 4 shows SEM images of a parylene-coated kapton substrate after a bending deformation. The surface crack may be clearly observed and, although it does not go deeper inside up to the substrate, it could propagate during the submersion period due to fatigue damage, as shown in the cross section inset achieved by focused ion beam (FIB). 

In this respect, the external coatings must have specific characteristics in order to protect the devices without affecting their flexibility: they should be conformal, lightweight, not fragile, non-porous, insulating and anti-biofouling [18,19,20,21,22]. 

Materials employed for insulating coatings of microscale electronic devices are mostly polymeric. The most frequently used materials are: (1) elastomers, (2) polyacrylates, (3) fluoropolymers, (4) poly-para-xylylenes (also known as parylenes).

Elastomers are thermosetting polymers with rubber-like properties, such as high stretchability and softness. The most widely used silicone-based elastomer is poly(dimethyl siloxane), PDMS, which has remarkable rheological, optical and non-toxic properties [23]. Generally, it is formed as a viscous bi-component thermosetting mixture between a siloxane-based oligomer and a curing agent, and applied by spin-coating [24], dip-coating [25] or spray-coating [26]. PDMS could also be incorporated, at different contents, into other polymeric coatings, such as polyurethane (PU), in order to enhance their antibiofouling properties, as reported in [27], or to make new composite materials [28].

Poly-(methyl methacrylate), PMMA, is one of the most common polyacrylates: it is a transparent thermoplastic polymer with noticeable mechanical and optical properties. It is generally used as a lightweight alternative to glass, but also for other various applications, such as inks and coatings, or for microfabrication processes as sacrificial layer. Coan et al [29] reported the development of composite coatings of PMMA with hexagonal boron nitride (hBN) as filler, for metal surface protection against corrosion. 

Fluoropolymers contain plenty of carbon-fluorine bonds which confer to the backbone a high chemical inertness against many solvents, acids or bases [30]. Some well-known examples are PVDF, or poly(tetrafluoroethylene), (PTFE). In particular, PVDF can be found commercially as extruded foils, pellets and micrometric powder as well, which can be incorporated in other polymeric matrices or dissolved in solvents.

Poly(-para-xylylenes) are thermoplastic semicrystalline polymers discovered by Michael Szwarc in the late 1940s and commercialized in 1965. These polymers are synthesized by chemical vapor deposition (CVD) and have very attractive properties among which low-adhesion coefficient at room temperature, and conformability to different types of substrates. The current method adopted to synthesize parylene is called the Gorham route and is a very efficient polymerization process, in fact it allows complete control of the deposition parameters: the process basically consists of pyrolizing the precursor dimer and polymerizing the resulting monomers during deposition onto the substrate [31,32,33]. Several different kinds of parylene may be synthesized, depending on the functional groups bonded to the backbone of the precursor (2,2-para-cyclophane): these substituents are not modified during the CVD process, making it possible to tailor chemical, mechanical, electrical and optical properties of parylene thin films and, therefore, to introduce diverse functionalities into the coated surfaces [31]. The range of application fields for parylene is wide: in particular, its insulating and moisture barrier properties make it suitable for protecting implanted biomedical micro-systems or devices in contact with water or wet environments [34,35,36]. 

Several works have been published about external polymeric coatings for insulation and barrier purposes [37,38,39]. Lewis and Weaver provide in [40] a review of thin-film permeation-barrier technologies for flexible organic light-emitting devices. Fredj et al. [41] studied the natural and artificial ageing of marine organic coatings. Deyab et al. [42] prepared a wax coating using waste materials (isolated microcrystalline waxes) to protect petroleum pipelines against corrosion in 0.6 M NaCl solution. The same author analyzed the effect of carbon nanotubes (CNTs) [43], newly synthesized titanium phosphates [44] or M-porphyrins [45] on corrosion protection of carbon steel coated by alkyd resin and tested after immersion in sodium chloride solution. Li et al. [46] performed accelerated soak tests to study the corrosion behavior and failure mechanisms of parylene-metal-parylene thin films. Davies and Evrard [47] studied polyurethanes for marine applications through accelerated tests at high temperatures. 

However, the application of protective coatings onto flexible micro-devices continuously moving underwater is a tricky issue, thus finding an optimal solution for guaranteeing at the same time (1) protection from the environment and (2) the best device performances is still an ongoing challenge. 

In this work, we have investigated the barrier behavior and surface properties of different polymeric coatings of the flexible piezoelectric transducers described above: parylene-C, poly-methyl methacrylate (PMMA) and poly-dimethyl siloxane (PDMS). These choices were made for the ease of applicability, conformability, low cost and compatibility with the flexible piezoelectric energy harvesters, since the external layers is crucial for the reliability of the device itself. The PDMS coating was used both in the neat form and mixed with a powder of PVDF. The combination of the conformability of the elastomer and the hydrophobicity and chemical inertness of the fluoropolymer was expected to confer higher water repellence to the coating, limiting water permeation in the device [48,49].

Furthermore, parylene was also designated as a suitable surface platform for evaluating the accumulation of microorganisms in a long-term period. Since previous articles in literature reported the surface functionalization of parylene by physico/chemical treatments [19,50,51], besides the pristine parylene-C (pC) coating deposited by CVD, surface-treated pC was taken into account: in particular, two surface treatments were adopted based on oxygen plasma etching and UV/ozonization.

The seawater absorption of coatings was analyzed by impedance spectroscopy (IS) measurements. The anti-corrosion properties of the coatings were tested by dynamic linear scanning voltammetry (LSV) measurements. Additionally, atomic force microscopy (AFM) measurements provided further information in terms of surface morphology. The barrier properties of the coatings and their reliability were therefore correlated to the higher or lower capability of retaining the formed biofilms: this evaluation was possible by estimating the amount of added microbial mass on the exposed surface through a laser Doppler vibrometer (LDV). 

## 2. Materials and Methods 

### 2.1. Materials

Parylene-C was provided by Specialty Coating Systems in form of dimer powders. PDMS (Sylgard 184 Silicone Elastomer) was supplied by Dow Corning Corporation in two compounds: a viscous uncured pre-polymer and a curing agent. PMMA 950 in Anisole e-beam resist (anisole 80–100%, PMMA 1–20%) was purchased from MicroChem Corp. Granular PVDF powder (average Mw ~ 534,000 by GPC) and 2-butanone (MEK) solvent were supplied by Sigma Aldrich. 

The piezoelectric devices employed in the present work were grouped into two categories: (i) AlN-based, and (ii) PVDF-based seaweed-shape transducers. The AlN flags were made using kapton HN (by Dupont in the form of 25 µm-thick foils) as substrate for thin film deposition. The commercial PVDF foils of the second groups of harvesters were provided by TE Connectivity’s Measurement Specialties (MEAS). 

AISI304 steel samples were selected as substrates for corrosion tests and were provided by RS Components. 

### 2.2. Fabrication of Flexible Transducers

The fabrication of the adopted transducers is reported, as for previous works [15,16].

#### 2.2.1. AlN-Based Transducers 

The kapton substrate was attached to a silicon wafer using polydimethyl siloxane (PDMS) spin-coated at 1000 rpm for 30 s and then cured at 90 °C for 15 min. A vacuum step was needed before curing to remove any residual air bubbles. The overlaying layers were deposited by reactive sputtering in a single run in order to minimize contaminations, with the following parameters: an AlN oriented growth-favoring interlayer (120 nm) and the first Mo layer (200 nm) were deposited in a single step and patterned by optical lithography and chemical etching. The AlN interlayer stemmed from a high-purity (99.9995%) Al target in a mixture of Ar (20 sccm) and N_2_ (20 sccm) gases with a total pressure of 2.8 × 10^−3^ mbar and with DC pulsed power supply of 750 W. The Mo electrode was deposited from a pure (99.95%) Mo target at room temperature, with a total pressure of 5 × 10^−3^ mbar in an Ar atmosphere (66 sccm) and with DC power of 400 W. The pattering of Mo bottom electrode and of AlN interlayer was performed by dry etching with inductively-coupled plasma-reactive ion etching (ICP-RIE) system: the gas mixture was made of BCl_3_ (45 sccm) and N_2_ (25 sccm) for Mo, and of BCl_3_ (100 sccm) and Ar (25 sccm) for the AlN. The applied power was 250 W to the platen and 600 W to the coil. The AlN piezoelectric layer (~1µm) and the second top Mo electrode (200 nm) were deposited in the same run: for the AlN film a high-purity Al target (99.9995%) was used with a gas mixture of N_2_ (20 sccm) and Ar (20 sccm) at a pressure of 2.8 × 10^−3^ mbar in DC pulsed mode with a frequency of 100 kHz and a power of 1000 W; for the Mo layer the same conditions as for the bottom electrode were applied. The chemical dry etching was performed by the ICP-RIE system under the same conditions as before. The chamber temperature during sputtering increased to ~70 °C and to ~165 °C for the Mo and AlN steps, respectively. Finally, the polymeric substrate was peeled off from the rigid wafer and the wire connections to the electrodes were made with a crimping tool.

#### 2.2.2. PVDF-Based Transducers

The PVDF foil was first cleaned with acetone and isopropanol before depositing the aluminum thin-film electrodes (200 nm) by thermal evaporation. The electrodes were patterned by using shadow masks made of adhesive kapton tape, whose shape was designed properly. Thermal evaporation allows to deposit the electrodes at a temperature of ~60 °C, which is lower than the Curie temperature of PVDF (~100–110 °C) and it doesn’t affect the piezoelectric feature of the functional layer. Finally, the wire connections were made with a crimping tool.

### 2.3. Preparation of Coatings

The methods for preparing the coatings are also reported in [52]. Moreover, in this work, the temperature was kept inside the range allowed by the device materials.

PDMS coating was prepared by mixing the pre-polymer with the cross-linker (10:1 wt), degassed for 30 min and applied on the whole devices by dip-coating. Finally, PDMS was cured in oven at 90 °C for 15 min.

PMMA-based coating was prepared by dip-coating the devices in the PMMA solution, then they were suspended in a heated oven at 90 °C for 1h for solvent evaporation. 

Coatings made of blends of PDMS and PVDF were obtained first mixing PDMS uncured pre-polymer with PVDF in a 3:1 (wt) ratio, then the mixture was heated in oven at 200 °C, above the PVDF melting point (177 °C), and stirred every 5 min. After cooling down at room temperature, the blend was mixed adding the curing agent (weight ratio 10:1 with respect to the PDMS). Finally, it was applied onto the devices by dip-coating and cured in oven at 90 °C for 15 min. 

Parylene-C deposition process was performed by a RT-CVD equipment (Specialty Coating Systems, PDS 2010 Labcoater system model). The powdered dimer vaporized at a temperature of ~100–150 °C and at a pressure of 1 torr to undergo a pyrolysis and be reduced in monomers; then, the polymerization of the gaseous monomers occurred at ~650–700 °C and 0.5 torr; the gas entered the deposition chamber at 20–25 °C and 0.1 torr and a conformal polymeric coating deposits on the substrate. An amount of 1 g of dimer powder yielded a deposited layer with thickness of 1μm and the process lasted approximately one hour.

### 2.4. Characterization and Reliability Tests

With the exception of parylene, the other coatings were applied on the devices by dip-coating so the control of thickness was subjected to uncertainties and non-uniformity. Different portions of the device were selected and the coating thickness was measured by means of a profilometer (Bruker Dektak Xt). 

#### 2.4.1. Surface Characterizations

The sample coatings were characterized in terms of wettability, through an OCA 15Pro Contact Angle Tool (DataPhysics), and surface morphology, by means of an atomic force microscope (CSI Nano-Observer AFM) in non-contact (resonant) mode, and a SEM (Helios NanoLab DualBeam, FEI). The reported values for contact angles were averaged over four independent measurements, whereas the roughness values stemmed from the averaging over a scanned area of 5 × 5 µm^2^. 

#### 2.4.2. Exposure to Seawater 

**Figure 5:**
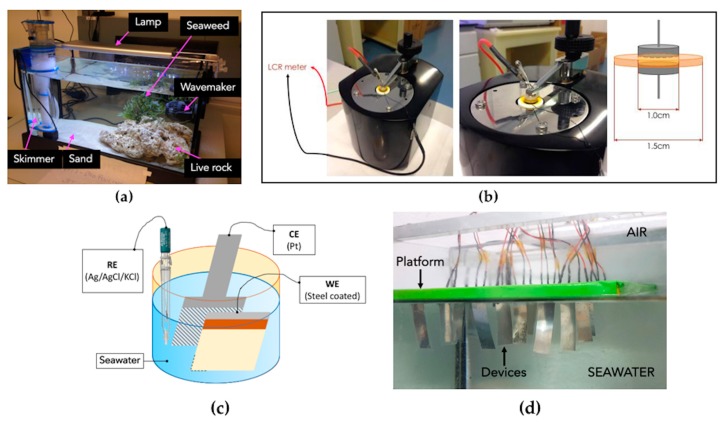
(**a**) Setup used to mimic the marine environment in the laboratory. (**b**) Setup used for the impedance spectroscopy (IS) measurements with the schematic of the samples adopted. (**c**) Schematic of the three-electrode cell used for the corrosion tests. (**d**) Floating platform for supporting the whole devices while being submerged in seawater for reliability tests.

The whole devices and some coated silicon substrates (three for each coating) were submerged in a tank (440 × 265 × 240 mm^3^) and filled with seawater which was aimed at reproducing the marine environment (Figure 5a). The natural seawater was collected from the Ionian Sea, with the following physico-chemical parameters: salinity 3.5%, pH 8, metals (Na 10,784 ppm, K 399 ppm, Mg 1294 ppm, Ca 4120 ppm, Si 2.9 ppm), halogenides (Br^−^/Cl^−^/F^−^ 19360/67.5/1.3 ppm), carbonates (HCO_3_^−^ 126 ppm), sulphates (SO_4_ 2712 ppm), and dissolved gases (O_2_ 7 ppm, N_2_ 12.5 ppm) [52]. 

A wave maker (Jebao RW4 Propeller Pump) was inserted in the tank to create sea waves and currents: in order to avoid the samples tumbling due to the water waves, a metallic grill was used as support; the coated rigid substrates were fixed on Petri dishes and placed in the grill, whereas the whole devices were made floating with a polystyrene supporting slab. A porous live rock was inserted to introduce natural bacteria, algae and invertebrates inside the artificial marine environment. Moreover, it plays the role of biological filter because it harbors aerobic and anaerobic nitrifying bacteria required for the nitrogen cycle. Furthermore, a natural seaweed was added. Its general function is to use waste products as nutrients and perform chlorophyllian photosynthesis, producing oxygen. In this study, it also served as a competing species for the development of bacteria and algae. A LED lighting system (Mini Lumina 30 Marine, Blau-aquaristic; 18 W; 10.000K white and 460 nm blue) was used to provide the solar light radiation, stimulating the growth of microorganisms. Finally, a skimmer (Scuma 0635 Int, Blau-aquaristic; 3.5 W) was installed to purify the environment keeping the water clear and limpid.

#### 2.4.3. Water Absorption Tests

The absorption of water into the devices mainly regards the polymeric layers. The conformity of the coatings is crucial because any possible asperity, uncovered area or inlet could lead to water permeation. Impedance spectroscopy (IS) was carried out to evaluate water absorption: circular samples (1.5 cm radius) of kapton coated with the selected polymers were submerged, then dried with nitrogen flow at regular time intervals. The impedances of the substrate/coating systems were collected by placing the samples between two metallic plates connected to an Agilent E4980A Precision Inductance, Capacitance, Resistance (LCR) meter (applying the same pressure for all samples) and by performing linear frequency sweeps.

#### 2.4.4. Corrosion Tests

Corrosion due to seawater penetration regards all the metallic portions of the devices, thus anti-corrosion tests were performed to evaluate the corrosion resistance of the coatings. The steel panels (4 × 5 cm^2^) were washed with acetone and isopropanol to remove organic contaminants before the application of coatings. The corrosion tests were carried out in a three-electrode cell (Figure 5c) consisting of coated steel samples as working electrode (WE), a 5 × 5 cm^2^ Pt mesh as counter electrode (CE) and an Ag/AgCl/sat-KCl electrode as a reference electrode (RE). Linear scanning voltammetry was performed at 1 mV/s to measure current and voltage using an AUTOLAB PGSTAT302N potentiostat, after 1h of stabilization to reach the equilibrium open circuit potential (OCP). 

#### 2.4.5. Piezoelectric Generation in the Long-Term Period

The PVDF-based devices were attached to a polystyrene foam slab and submerged in the tank filled with seawater (Figure 5d): the slab served as a supporting platform to let the devices float firmly on the water. In this way, the supporting platform is always in contact with the water surface although the continuous evaporation of water, keeping the same initial conditions. The measurements were performed by displacing the samples at a proper distance, using a linear micro-actuator (Actuonix Motion Devices Inc., L16-R, 100 mm, 35:1, 6vdc) and the output signal generated by the oscillation of the devices (open circuit voltage) was detected and collected with an oscilloscope (Tektronix MDO 4104-3).

#### 2.4.6. Surface Treatments of Parylene Surface

Wettability and morphology studies were performed on treated parylene C coatings in order to evaluate qualitatively the long-term microbial adhesion. Two different treatments were applied: oxygen plasma and ultraviolet light irradiation with addition of ozone. Oxygen plasma treatment was performed by a low-pressure plasma system NANO (Diener electronic Plasma Surface Technology GmbH & Co. KG), with two different RF powers in order to compare the resulting modified morphologies, i.e., 100 W and 300 W, keeping the process time (20 min) unchanged. UV/ozone treatment was performed by a UV Ozone Cleaner ProCleaner Plus (BioForce Nanosciences) for 20 min. The two treatments were overlapped in order to evaluate the effects of each single treatment. As previously demonstrated [50], the etching effect of oxygen plasma treatment on polymeric substrates induces a structure with nanosized thread-like features with a relatively high roughness. The lower the dimensions of asperities (roughness), compared to the dimensions of air bubbles in water, the less likely these air bubbles remain trapped among the asperities, thus the wider the contact surface/water and the more favored the water absorption. For these reasons, the UV/ozone treatment was superimposed to the oxygen plasma with the intention of making the sample surfaces less rough.

Non-treated samples, samples treated with O_2_ plasma, and samples treated with both the O_2_ plasma and UV/ozone treatments, were compared. The flexible kapton/pC structures were characterized after submerging them in the tank: the characterization tests were carried out at three different periods of time (15, 25, 35 days), after drying the samples in a desiccator.

The laser Doppler vibrometer (LDV-MSA-500, Polytec), equipped with HeNe laser light source, was employed to investigate the frequency response of the pC-coated kapton samples evaluating the out-of-plane deflections by a non-contact measurement. The experimental setup included also an electromechanical shaker (4819, Bruel & Kjaer) aimed at providing the mechanical vibrations in a range of 0–18kHz. The samples were previously cut and shaped in the form of cantilevers (10 × 5 mm^2^) by an automatic cutting plotter (Graphtec Cutting Plotter CE6000-40).

## 3. Results

This work was aimed at evaluating the protective and barrier behavior of different selected polymeric coatings for flexible piezoelectric energy harvesting devices in liquid flows, in particular in a seawater environment. The application and the type of micro-devices required specific characteristics for the coatings, i.e., flexibility, conformability, ease of application, besides properties related to the insulating, waterproof and barrier behavior. 

As already discussed, parylene-C, PMMA, PDMS and PDMS-PVDF were selected as coating materials to be tested, each characterized by different thicknesses: 2.7 µm for parylene, ~3 µm for PMMA, ~100 µm for PDMS and PDMS-PVDF. Differently from the other coatings applied by dip-coating, parylene C was deposited by CVD, so the thickness of the resulting coating was much more controllable. 

### 3.1. Surface Characterizations

**Figure 6:**
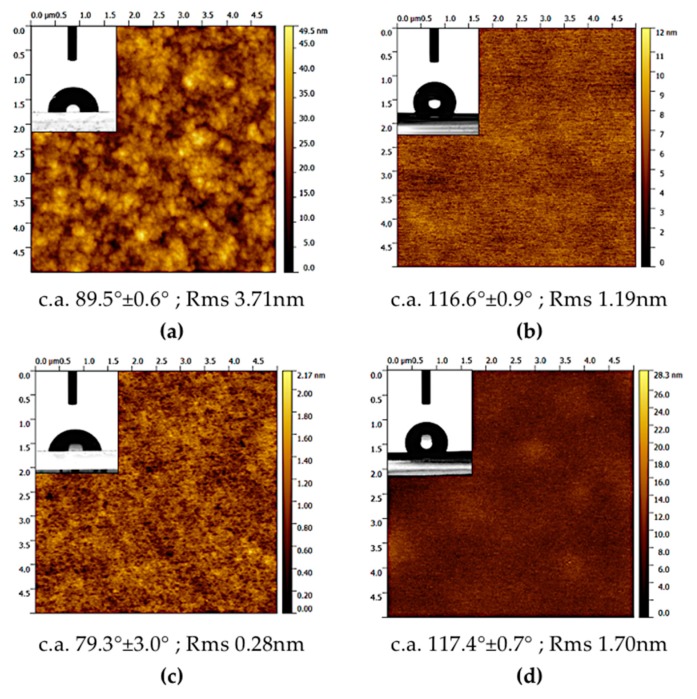
Atomic force microscopy (AFM) topography images (scanned area: 5 × 5 µm^2^) for the examined coatings: (**a**) parylene-C, (**b**) poly-dimethyl siloxane (PDMS), (**c**) poly-methyl methacrylate (PMMA), (**d**) PDMS- and poly-(vinylidene fluoride) (PVDF). The vertical color bar reports the roughness in nm. The insets show the results of contact angle measurements.

The AFM micrographs and the results of wettability measurements are reported in Figure 6. 

Parylene C (pC) exhibited higher roughness (3.71 nm) than PMMA (0.28 nm) and PDMS-based coatings which present comparable values, i.e., 1.19 nm (PDMS), 1.70 nm (PDMS/PVDF). Concerning the wettability characteristics, parylene and PMMA showed contact angles of 89.5° ± 0.6° and 79.3° ± 3.0°, respectively; PDMS-based coatings present c.a. values of 116.6° ± 0.9° (PDMS) and 117.4° ± 0.7° (PDMS/PVDF): these values indicate that PMMA has a moderately hydrophilic character, whereas the other coatings exhibit more hydrophobicity, apart from parylene which may be considered as neutral; the incorporation of PVDF into the PDMS slightly increased the contact angle.

### 3.2. Water Absorption Tests

**Figure 7:**
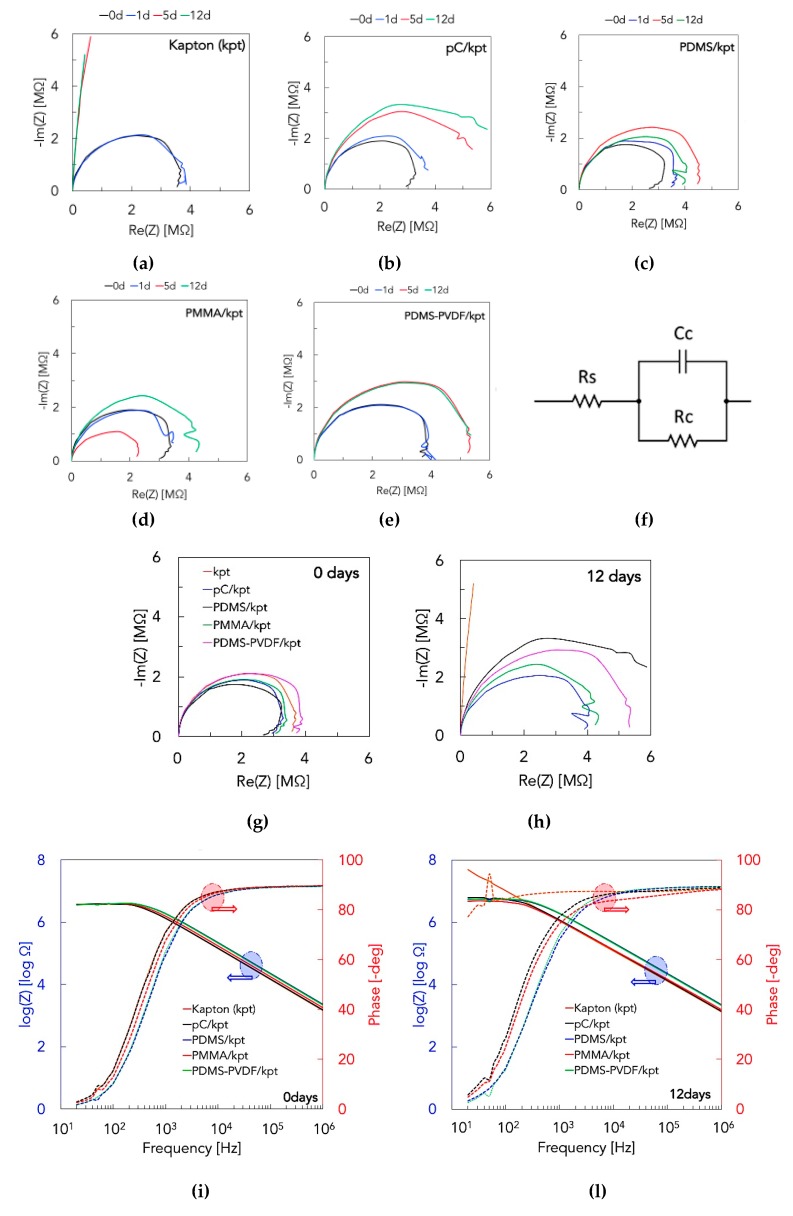
Nyquist plots resulting from IS measurements (frequency sweep in the range 20Hz–1MHz), for the kapton substrate (**a**) and for the coated substrates: (**b**) parylene-C, (**c**) PDMS, (**d**) PMMA, and (**e**) PDMS-PVDF. In each plot, the curves correspond to different periods of exposure to seawater, i.e., 0, 1, 5, 12 days. (**f**) The equivalent circuit model used to fit IS data: R_s_ is the resistance of wires and electronic parts; R_c_ and C_c_ are the resistance and capacitance of the coating. (**g**,**h**) Nyquist plots for the coated substrates, at 0 (**g**) and 12 (**h**) days of exposure to seawater. (**i**,**l**) Bode plots for the coated substrates, at 0 (**i**) and 12 (**l**) days of exposure to seawater.

The IS frequency-sweep analyses performed with the setup shown in Figure 5b on the submerged samples yielded the Nyquist plots reported in Figure 7a–e. The shape of the Nyquist curves is in general semi-circular and the magnitude of the vector connecting the axis origin with each point of the curves gives the values of impedance for different frequencies. Therefore, a curve stretched upward indicates that the imaginary part of impedance (reactance) of the system is higher.

The barrier behavior of the coatings is evident when comparing them with the uncoated substrate. As can be seen from the Nyquist plots, the curves in the latter case become almost linear after 5 and 12 days of exposure to seawater, whereas for all the other samples the curves keep a quasi-circular shape. In other words, with the passing of submersion time, the reactance of the system gets higher because of the absorption of water molecules with high dielectric constant, but to a much lower extent when the substrate is coated. The absorbed water molecules eventually dissociated, and ions are responsible of a change also in the real part with exposure time. The behavior of electrical resistance and reactance of the coating/substrate systems is a result of different phenomena: (1) the water molecules uptake by absorption, (2) the different swelling degree of the coatings, (3) the damage/delamination of the coatings allowing seawater permeation, (4) the dissociation of absorbed water molecules and permeation of solvated ions. The presence of salty water, or dissociated water molecules or solvated ions in the coatings induces a decrease in the electrical resistance, while the absorption of water molecules (with high dielectric constant) and the swelling behavior lead to an increase of the dielectric constant of insulating materials [53,54,55,56,57]. From the analysis of the IS plots it can be deduced that for the selected coatings the latter phenomenon is prevalent. Bode plots reported in Figure 7i,l highlight the frequency behavior of the impedance and phase angle for the tested substrates. The general trend is a decrease in impedance and an increase in phase with increasing frequency due to a more dominant capacitive behavior. In summary, from the Nyquist plots (Figure 7g,h) and the Bode plots (Figure 7i,l), at 0 and 12 days of exposure to seawater, it can be deduced that the protective action of the different coatings is quite comparable, and a further quantitative analysis was necessary: in Table 1, the coating resistance (R_c_) and capacitance (C_c_) are reported as results of the fitting of IS data in ZView® software, for which the equivalent circuit model in Figure 7f was adopted for the coating/substrate system: generally, the fitted data match the experimental, with an average error of 5.0%, and higher resistances or lower capacitances indicate a more protective ability of the coatings; after 12 days of exposure to seawater, parylene-C shows the best behavior.

### 3.3. Corrosion Tests

**Figure 8:**
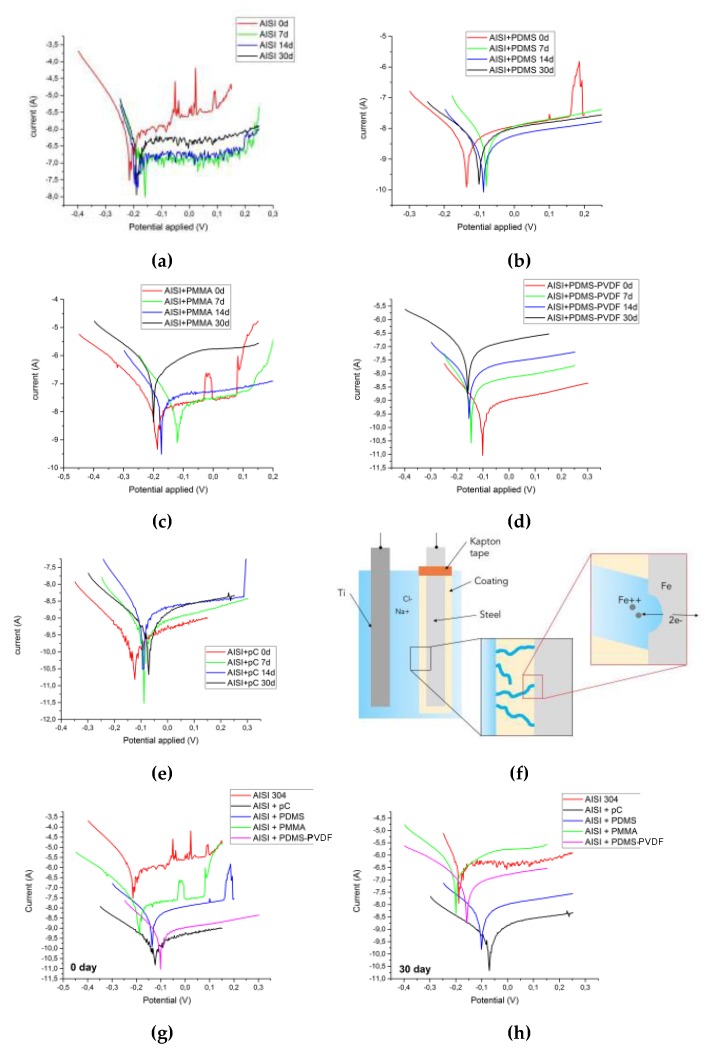
Evan diagrams displaying the Tafel plots resulting from corrosion tests for the pristine steel samples (**a**) and for the coated samples: (**b**) PDMS, (**c**) PMMA, (**d**) PDMS-PVDF, (**e**) parylene-C. The curves in each plot correspond to different periods of submersion, i.e., 0, 7, 14, and 30 days. The *y*-axis in the plots is in logarithmic scale. (**f**) Corrosion mechanism of steel samples due to seawater absorption and permeation. (**g**,**h**) Tafel plots for the differently coated steel samples, at 0 (**g**) and 30 (**h**) days of exposure to seawater.

The corrosion tests on the steel samples with the selected coatings yielded the current–voltage curves (Tafel plots) in the Evans diagrams, as reported in Figure 8, which provided more details about the anti-corrosive properties of the coatings: the less porosities and better conformal coverage, the more effective the coating.

Figure 8 indicates that the current passing through the seawater solution, (when the WE is pristine steel), is higher whereas it is much lower when steel is coated. As matter of fact, a coating is more effective if the allowed current level is lower.

As illustrated in Figure 8f, the reason for a non-zero current passing through the coating regards the presence of defects across its thickness: these defects, which include porosities, delamination and cracks, allows the direct contact of seawater with the underlying steel, closing the circuit. 

The sparks on the anodic polarization curves are due to noisy current fluctuations which are unavoidable and cannot be eliminated [58]. Since the medium in the electrochemical three-electrode cell consists of seawater (basically water and NaCl) with pH slightly above 8, steels show passive behavior but Cl^−^ ions continuously disrupt the passivation film which tries to re-form. Another mechanism could involve the formation of OH* radicals at the metal surface, which attack the polymer leading to deterioration [35]. Therefore, the alternating process of localized formation and breaking of the passive film results in current fluctuations in the Tafel plots: this behavior is more evident for uncoated steel samples whose curves are overall quite noisy. In addition, the sparks at the edge of the curves (i.e., in potentials far from the corrosion potential), even for coated samples, may be caused by bubbling of gases produced by electrolysis of water.

The Tafel plots for the studied coatings were overlapped and compared for the same period of time, i.e., at 0 and 30 days of submersion, as shown in Figure 8g,h. It is worth noticing that:

1. At 0 days the best coatings are parylene-C, PDMS and the combination PDMS-PVDF, whilst the worst is PMMA, in terms of insulation and anti-corrosive behavior;
2. At 30 days the best coatings are parylene-C and PDMS, whereas PDMS-PVDF gets worse perhaps owing to inhomogeneity; PMMA confirms to be the worst coating.

Electrochemical kinetic values (corrosion potential E_corr_ and corrosion current density j_corr_) were calculated from the intersection of coordinates of Tafel plot [59] and are presented in Table 2. The protection efficiency (η_j_%) of the coatings are also reported according to calculations made using the following formula [60,61]:

η_j_% = [1 − (j_corr_/j^0^_corr_)] × 100 (1)

where j^0^_corr_ and j_corr_ are the corrosion current densities in the absence and presence of coatings, respectively.

**Figure 9:**
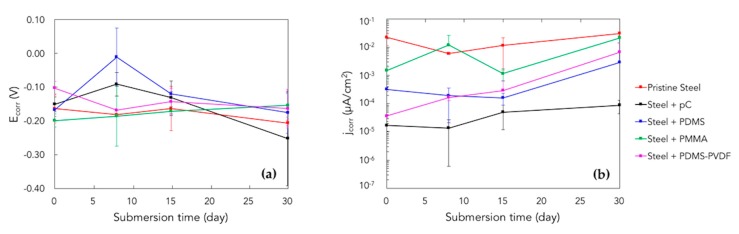
Corrosion potential (**a**) and corrosion current density (**b**) vs. submersion time, for the pristine steel samples and the selected coatings.

The time variation of corrosion potential and current density during submersion is shown in Figure 9 for the pristine steel and the coated samples.

From these results it is evident that initially the protection efficiency increases in the following order: PMMA < PDMS < PDMS-PVDF < pC, whereas, for long-term periods: PMMA < PDMS-PVDF < PDMS < pC.

### 3.4. Piezoelectric Generation in the Long-Term Period

**Figure 10:**
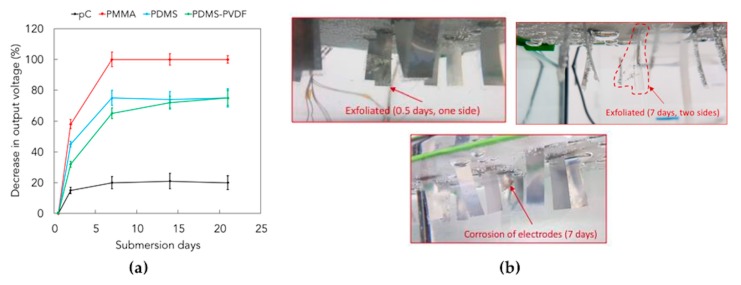
(**a**) Decrease (%) in output voltage of piezoelectric PVDF-based transducers due to prolonged exposure to seawater. (**b**) Exfoliation of PMMA coatings after exposure to seawater.

The measurements of the open circuit voltage generated by the submerged devices yielded the plot reported in Figure 10a, as percentage decrease of the output signal. All curves are characterized by a plateau indicating that the piezoelectric performance remains constant in continuous use applications, even if with remarkable differences among the coatings. These results confirm those obtained in corrosion tests, i.e., a complete loss of signal is observed after 7 days with PMMA coating, exhibiting the worst performances (exfoliation can be observed in Figure 10b). PDMS and PDMS/PVDF coatings are substantially equivalent showing a stable voltage loss of 70%, while a voltage loss of less than 20% is observed when pC is used. These results provide a direct correlation among device performance and mass transport properties of the coatings. 

### 3.5. Observation of Microbial Adhesion on Pristine and Treated Parylene

This study was also focused on evaluating the possibility of modifying the surface properties of parylene-C thin films in order to use them as protecting materials against marine biofouling in electronic devices. Both the treatments adopted on the polymeric surfaces bring about two kinds of effects due to the simultaneous occurring of different processes [62]: (1) a physical effect, strictly related to the etching and sputtering of the polymer owing to the reactions of oxygen atoms with the surface carbon atoms and to the impingement of plasma ions onto the surface, leading to a change in morphology; (2) a chemical effect, which is provided through the incorporation of hydrophilic and oxygen-rich functional groups on the surface, and through the breakage of organic bonds promoted by the UV radiation emitted by the plasma or by the UV source. Plasma treatments, as well as UV/ozone, are usually used for cleaning, surface activation, deposition and etching, and are typically employed for modifying the chemical and physical surface properties of polymers [63].

**Figure 11:**
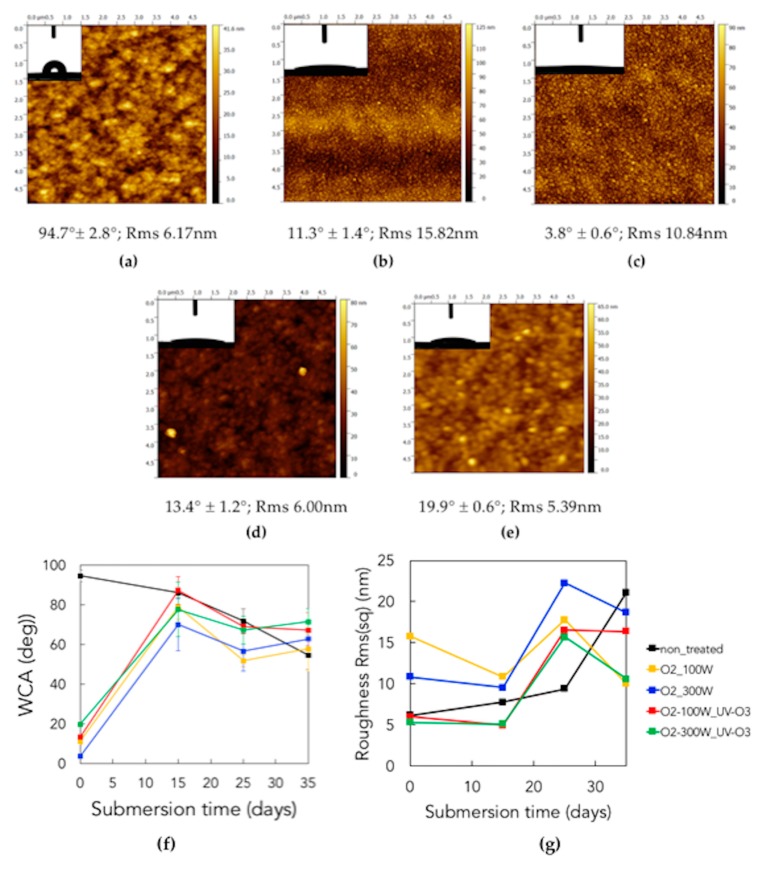
**Seawater contact angle** (WCA) measurements and AFM topography micrographs for non-submerged samples: non-treated (**a**), treated with oxygen plasma at 100 W (**b**) and 300 W (**c**), treated with oxygen plasma at 100 W, 300 W and UV/ozone (**d,e**). The scanned area for all AFM images was 5 × 5 µm^2^. (**f**) Seawater contact angle, WCA, and (**g**) roughness vs. submersion time: each curve corresponds to one specific treatment; each point in the roughness plot is the result of averaging on the scanned area.

Seawater contact angle (WCA) measurements, as shown in Figure 11 (compared to Figure 6), confirmed that the oxygen plasma surface treatment increases the hydrophilic character of the sample surface with respect to non-treated surface (94.7° ± 2.8°), and this also occurs to a larger extent when a higher generator power is set: in fact the recorded WCA is 11.3° ± 1.4° for 100 W, and 3.8° ± 0.6° for 300 W. On the other hand, the UV/ozone treatment, performed subsequently to the oxygen plasma treatment, counter-intuitively results in a slight decrease in hydrophilicity (maintaining however a hydrophilic character, with WCA values of 13.4° ± 1.2° and 19.9° ± 0.6° for 100 W and 300 W powers of oxygen plasma treatment, respectively), revealing a counteraction between the two treatments.

The wettability properties are strictly correlated to the surface roughness (Rms) measured by AFM and reported in Figure 11. The recorded roughness of non-treated samples is 6.17 nm, whereas the oxygen plasma treatment increases the roughness, with the formation of thin threadlike structures, at a higher extent for lower powers (15.82 nm for 100 W, 10.84 nm for 300 W), due to the stronger physical etching provoked by plasma, with a resulting higher etching rate. 

The blurry aspect of AFM images of samples treated with oxygen plasma and UV/ozone can be correlated to the chemical modification caused by the UV/ozone treatment. This process increases the amount of oxygen-containing reactive species on the surface, which are very likely to electrostatically interact with the surrounding water vapor molecules. Furthermore, the UV/ozone treatment causes a reduction of roughness (6.00 nm and 5.39 nm for 100 W and 300 W of oxygen plasma treatment, respectively) with less sharp asperities. This result can be explained by a more homogeneous levelling action than that of oxygen plasma. The decrease in roughness is also consistent with the decrease in hydrophilicity, according to Wenzel’s wettability model [64]. 

During submersion the non-treated sample became more hydrophilic (54.8° ± 7.3° at 35 days) and the others became more hydrophobic (in the range between 57° and 72°). However, the final values of water contact angle were quite similar, as shown in Figure 11f. This is an indirect confirmation that the long-term growth of biofilm induces a common wetting behavior. AFM results for the roughness evolution of the submerged samples are plotted in Figure 11g. As can be seen, microorganisms colonize randomly the devices surface, producing extracellular matrix, thus the roughness values provide information on compactness, homogeneity and regularity of the biofilm. After an initial increase in roughness (up to 25 days), the biofilm becomes more and more populated and composed of microbial aggregates rather than isolated cells, whose effect is a self-levelling action, with a reduction in roughness (after 30 days) [65]. The only exception is given by the non-treated sample, allegedly because the microorganisms attach on the surface but, with the passing of time and the progressive scarcity of nutrients, their bioadhesive strength weakens. Since there are much less asperities than for the other samples, they are washed off more easily, leaving a much more non-homogeneous (thus rougher) surface after 30 days.

**Figure 12:**
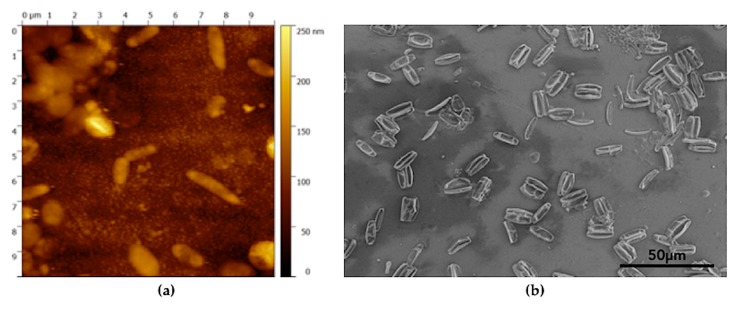
(**a**) AFM image with bacteria (~1 µm). (**b**) SEM micrograph with diatoms (~10 µm).

AFM and SEM micrographs in Figure 12 highlight the presence of microorganisms on the sample surface, in particular a large number of bacteria (with dimensions in the range of 1 ÷ 2 μm), and diatoms Bacillariophyceae (6 ÷ 10 μm).

**Figure 13:**
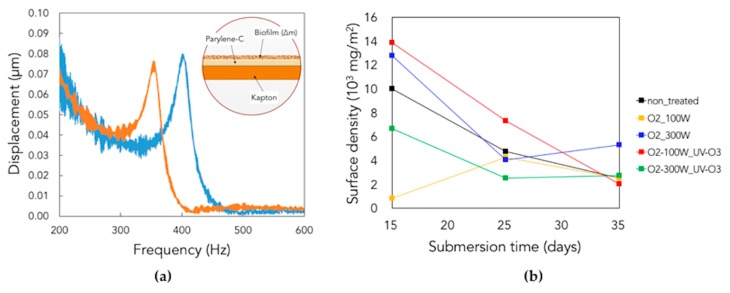
(**a**) Shift in resonance frequency due to the growth of biofilm; the inset shows the model used for the simulations. (**b**) Biofilm surface density vs. submersion days: each curve corresponds to a specific surface treatment.

Finally, as a further proof of the microbial adhesion, LDV analyses highlighted the change of the resonance frequency of the submerged samples with respect to the non-submerged ones (Figure 13a), caused by the growth of biofilm on their surfaces. Finite element method (FEM) simulations (Eigenfrequency analysis, COMSOL multiphysics) were needed to derive the mass surface density of the grown biofilm from the experimental data on resonance frequencies. A simple 3D model was built on (a bi-layer pC/kapton substrate with a variable added biofilm mass on the top of it; see the inset in Figure 13a), showing that the experimental frequencies are in good agreement with the computational values allowing to derive and calculate the biofouling mass added on the sample surfaces: Table 3 summarizes the experimental and computational resonance frequencies, with the corresponding biofilm added mass, for the differently treated samples.

At longer times, a common general decrease of the surface mass density, associated with an increase of resonance frequency, was detected on non-treated and treated substrates due to the degradation of biofilm and the death of microorganisms (Figure 13b).

## 4. Conclusions

In this study protective, barrier and anti-corrosive properties of different polymeric coatings for applications in marine environments, were compared. The final specific use of these thin films consists of employing them as conformal coatings for insulating flexible micro-devices adopted for harvesting mechanical energy from fluid-induced oscillations. 

The desired application poses several challenges and issues to be addressed, i.e., the risk of short-circuit, the corrosion of metal electrodes, the water absorption and permeation into the devices leading to delamination or damages, the adhesion of microorganisms on the device surfaces. Therefore, the selection of the coatings to be tested was made in order to satisfy several requirements, such as flexibility, conformability, ease of application, insulating and barrier properties. 

IS analyses, corrosion tests and the measurements of the piezoelectric signal for coated submerged devices revealed that parylene-C provides the best protecting, insulating and adhesion properties among the other coatings, even though it is susceptible to surface crack formation more than elastomers (PDMS-based coatings), because of its thermoplastic semi-crystalline character. Definitely, PMMA is the worst alternative mainly because it easily undergoes exfoliation during exposure to seawater.

Therefore, parylene was also used to observe qualitatively the accumulation of microorganisms and formation of biofilm on the surface of submerged devices: different surface treatments were adopted as well to modify the morphology and wettability of parylene in order to correlate microbial adhesion changes in the long-term period. The surface treatments selected for this purpose comprised an oxygen plasma treatment and a UV/ozone treatment. The reduction in the surface mass density of the grown biofilm, for both treated and non-treated substrates, revealed a loss of adherence for the microorganisms which is ascribed to the local scarcity of nutrients and to the protective action of parylene-C thin film: thus, together with its strong chemical and moisture resistance, this further property of parylene-C makes it more attractive as a conformal passivation coating on polymeric substrate materials and flexible micro-devices.

## Figures and Tables

**Table 1:** Impedance parameters resulting from fitting the IS data for the uncoated and coated kapton substrates (1.5 cm diameter).

Submersion Days	Samples	R_c_ [10^6^ Ω]	C_c_ [10^−10^ F]
0	Kapton (kpt)	3.5276	1.0910
	pC/kpt	3.9573	1.0650
	PDMS/kpt	3.7389	0.7080
	PMMA/kpt	3.6062	0.8885
	PDMS-PVDF/kpt	3.7677	0.7325
12	Kapton (kpt)	- (low)	1.3430
	pC/kpt	6.5529	1.2230
	PDMS/kpt	4.0184	0.7404
	PMMA/kpt	4.3604	1.1510
	PDMS-PVDF/kpt	5.5225	0.7552

**Table 2:** Electrochemical parameters and the corresponding inhibition efficiency for steel coated with the selected coatings.

Submersion Days	Samples	E_corr_ (vs. SCE) [V]	j_corr_ [µAcm^−2^]	η_j_ [%]
0	Pristine AISI314 steel	−0.1635 ± 0.0426	(2.27 ± 1.14) × 10^−2^	-
	pC	−0.1511 ± 0.0217	(1.70 ± 0.65) × 10^−5^	99.9
	PDMS	−0.1677 ± 0.0192	(3.21 ± 0.58) × 10^−4^	98.6
	PMMA	−0.1990 ± 0.0191	(1.49 ± 0.80) × 10^−3^	93.4
	PDMS-PVDF	−0.1030 ± 0.0202	(3.67 ± 0.45) × 10^−5^	99.8
30	Pristine AISI314 steel	−0.2065 ± 0.0084	(3.10 ± 0.47) × 10^−2^	-
	pC	−0.2522 ± 0.1398	(8.84 ± 4.37) × 10^−5^	99.7
	PDMS	−0.1759 ± 0.0606	(2.90 ± 4.43) × 10^−2^	90.7
	PMMA	−0.1542 ± 0.0349	(2.15 ± 0.74) × 10^−2^	30.6
	PDMS-PVDF	−0.1635 ± 0.0572	(6.75 ± 7.96) × 10^−2^	78.2

**Table 3:** Results of finite element method (FEM) simulations.

Submersion Days	Samples	Length (mm)	Width (mm)	Exp f_res_ (Hz)	FEM f_res_ (Hz)	Biofilm Mass (10^−5^ g)
0	-	5.5	3.0	230.5	232	-
15	NT	6.0	3.0	201.25	201.2	18
	O_2__100W	6.0	3.0	225	225.5	1.5
	O_2__300W	6.0	3.0	208.28	208.4	23
	O_2_-100W_UV-O_3_	6.0	3.0	196.72	196.8	25
	O_2_-300W_UV-O_3_	6.0	3.0	226.25	226.1	12
25	NT	4.5	2.2	423.125	423.47	4.75
	O_2__100W	4.5	2.2	400.94	400.17	4.25
	O_2__300W	4.5	2.2	398.75	398.5	4.00
	O_2_-100W_UV-O_3_	4.5	2.2	354.69	354.9	7.25
	O_2_-300W_UV-O_3_	4.5	2.2	423.44	424.1	2.50
35	NT	4.5	2.2	408.75	408.9	2.50
	O_2__100W	4.5	2.2	430.94	431.1	2.50
	O_2__300W	4.5	2.2	406.41	406.8	5.25
	O_2_-100W_UV-O_3_	4.5	2.2	407.81	407.8	2.00
	O_2_-300W_UV-O_3_	4.5	2.2	427.03	427	2.75
